# Evaluating the potential of fermented bakery by-products as a replacement for corn gluten feed in cattle diets to suppress methanogenesis and alter rumen fermentation in growing Holstein bulls

**DOI:** 10.3389/fmicb.2025.1485688

**Published:** 2025-03-20

**Authors:** Xuanxuan Pu, Wanqian Zhang, Fan Yang, Xiumin Zhang, Rong Wang, Qiushuang Li, Xingze Yang, Daliang Cai, Jiabin Huo, Xuezhao Sun, Zhiliang Tan, Bo Lin, Min Wang

**Affiliations:** ^1^CAS Key Laboratory for Agro-Ecological Processes in Subtropical Region, National Engineering Laboratory for Pollution Control and Waste Utilization in Livestock and Poultry Production, Institute of Subtropical Agriculture, the Chinese Academy of Sciences, Changsha, Hunan, China; ^2^College of Animal Science and Technology, Tarim University, Alar, Xinjiang, China; ^3^Key Laboratory of Livestock and Forage Resources Utilization around Tarim, Ministry of Agriculture and Rural Affairs, Alar, Xinjiang, China; ^4^College of Animal Science and Technology, Guangxi University, Nanning, Guangxi, China; ^5^The Hubei Provincial Key Laboratory of Yeast Function, Yichang, Hubei, China; ^6^AgResearch Limited, Grasslands Research Centre, Palmerston North, New Zealand

**Keywords:** fermented bakery by-products, nutrient digestibility, dissolved methane, rumen fermentation, bacteria

## Abstract

Both corn gluten feed and bakery by-products are important alternative concentrate feedstuffs for ruminants. Bakery by-products, which are rich in ether extract (EE) and starch, have the potential to be utilized as concentrate feedstuffs for ruminants, with a capacity to reduce ruminal methanogenesis. In the study, fermented corn gluten feed (FCG) and fermented bakery by-products (FBP) were mixed with other feedstuffs to formulate FCG and FBP diets, respectively. Twenty growing Holstein bulls, weighing 241 ± 10.5 kg, were randomly assigned to one of two dietary treatments: FCG or FBP diet. The aim was to investigate effects of replacing FCG with FBP feedstuff on nutrient digestibility, ruminal fermentation, ruminal microbiota, and methanogenesis. Results showed that the bulls feeding FBP diet had greater starch intake (*p* < 0.01) and digestibility (*p* = 0.04), EE intake (*p* < 0.01) and digestibility (*p =* 0.01), molar proportion of ruminal propionate (*p* < 0.01), while lower crude protein (CP) (*p* < 0.01) and neutral detergent fiber (NDF) digestibility (*p =* 0.01), ruminal dissolved methane concentration (*p* = 0.02), percentage of ruminal acetate (*p* < 0.01) and butyrate (*p* < 0.01), and the ratio of acetate to propionate (*p* < 0.01), in comparison with those feeding FCG diet. Further investigation on the bacterial community indicated that feeding the FBP diet had greater abundance of *Succiniclasticum* (*p* = 0.02), *Megasphaera* (*p* < 0.01), *Lachnospiraceae_unclassified* (*p* < 0.01) and *Lachnospira* (*p* < 0.01), while lower abundance of *Christensenellaceae_R-7_group* (*p* < 0.01), *Ruminococcus* (*p* < 0.01) and *NK4A214_group* (*p* = 0.01). The increases in EE and starch intakes after the substitution of FCG by FBP feedstuff alter fermentation rumen pathway from acetate to propionate production through enriching the propionate producers with net hydrogen incorporation, and reduced ruminal methanogenesis.

## 1 Introduction

By-products that are high in ether extract (EE) and starch offer strategies to meet the nutrient requirements of growing ruminants, while reducing food competition with humans (Humer et al., 2018; Kaltenegger et al., 2020). Bakery by-products, which are leftovers from food processing plants or bakeries, are available in large quantities and have the potential to be used as concentrate feed for ruminants. Recently, a new segment of the feed industry based on bakery by-products has emerged (Humer et al., 2018).

Corn gluten feed is a by-product of corn starch production via the wet milling process and includes bran and steep (Jiao et al., 2022). It is rich in digestible corn fiber and protein, and has been widely used as a concentrate feedstuff for ruminants (Bernard et al., 1991; Siverson et al., 2014). While corn gluten feed has been reported to have no negative effects on the productive performance of dairy cows or steers, replacing grains with corn gluten feed might result in greater methane production due to its high fiber and low starch content.

Archaeal methanogens use hydrogen as an energy source for their own growth and are the primary hydrogen users in the rumen with methane as end products (Wang et al., 2013). Methane production represents a decrease in energetic efficiency, contributes to greenhouse gas emissions and exerts great impact on global climate change (Li et al., 2022; Wang et al., 2022; Wang et al., 2024). Higher levels of starch in the diet would degrade more rapidly into volatile fatty acids (VFAs), leading to a lower ruminal pH, which can inhibit the metabolic activity and numbers of methanogens (Van Kessel and Russell, 1996; Qiu et al., 2023). Additionally, starch fermentation in the rumen favors propionate production, which serves as a hydrogen sink (Hegarty, 1999; Wang et al., 2016a,b). Apart from starch, higher fat concentration in the diet can decrease ruminal methanogenesis by reducing the metabolic activity and numbers of protozoa and/or enhancing hydrogen utilization through biohydrogenation (Henderson, 1973; Zhang et al., 2019). Therefore, it is speculated that high levels of starch and EE in the bakery by-products could reduce ruminal methanogenesis when used in the ruminant diets.

The high sugar and moisture contents of bakery by-products or corn gluten feed make them prone to spoilage, which can be mitigated through solid-state fermentation. Therefore, bakery by-products (a mixture of bread, rolls, biscuits and cakes) and corn gluten feed were fermented to obtain fermented bakery by-products (FBP) and fermented corn gluten feed (FCG) feedstuff, respectively. The substitution of FCG with FBP feedstuff could lead to changes in both ruminal fermentation end-products and the microbial community in bulls, primarily due to an increase in starch and EE content or a reduction in fiber in the diets. It was hypothesized that the ruminal methanogenesis could be suppressed by the greater inclusion of starch and EE following the replacement of FCG with FBP feedstuff. To test this hypothesis, the objective of the study was to compare the effects of FCG feed and FBP on methanogenesis, fermentation pathways, and the bacterial community in the rumen of growing Holstein bulls.

## 2 Materials and methods

All animals involved in the experiment were cared for in accordance with the Animal Care and Use Guidelines of the Animal Care Committee, Institute of Subtropical Agriculture, the Chinese Academy of Sciences, Changsha, China, with all animal experimental procedures approved by the committee (approval number ISA-WM-202101).

### 2.1 Preparation of FBP and FCG

Bakery by-products used in the present study comprised a mixture of 600 g/kg bread crumbs, 200 g/kg cookie crumbs and 200 g/kg cake crumbs, which were sourced from Munters new Co., Ltd (Nanning, China). Semi-dried corn gluten feed was sourced from Nanning Junwu Biological Technology Co., Ltd (Nanning, China). Both of these feedstuffs were sprayed with distilled water to adjust the moisture content to approximately 330 g/kg on a dry matter (DM) basis. They were then fermented in non-anaerobic bags (each weighing approximately 50 kg per bag) at 25°C for 15 d. Samples of FCG and FBP were dried at 65°C for subsequent chemical composition analysis (Table 1).

**Table 1:** Chemical composition of fermented corn gluten feed (FCG) or fermented by-products (FBP) feedstuff.

Items^a^	Feedstuffs	
	FCG	FBP
DM, g/kg	944	965
Substrate on a DM basis, g/kg DM		
OM	810	963
CP	198	112
NDF	335	160
EE	47	153
Starch	97	356
GE, MJ/kg DM	15.6	20.8

a DM, dry matter; OM, organic matter; CP, crude protein; NDF, neutral detergent fiber; EE, ether extract; GE, gross energy.

### 2.2 Animals, diets and experimental design

Twenty healthy growing Holstein bulls, weighing 241 kg ± 10.53 kg, were employed and randomly assigned to one of the two dietary treatments, with 10 bulls per group. The treatments consisted of diets containing either FCG or FBP feedstuff. The FBP diet was formulated to completely replace FCG by FBP feedstuff. Both diets were designed to meet appropriately 1.2–1.3 times the energy and protein requirements of growing beef bulls (Table 2), and were offered as total mixed rations which were mixed in the TMR machine (9JGW-5A). All bulls were housed in individual pens, and fed equally at 08:00 and 16:00 daily. Feed offered and refused was weighed and recorded each day. The experiment lasted for 67 days, including a 7-d period for environmental and dietary adaption and a 60-d period for measuring animal performance and collecting samples. Sampling was conducted on the last 7 d of the experiment, consisting of a 5-d for digestibility measurements and a 2-d for the collection of rumen contents.

**Table 2:** Ingredients and chemical composition of diets containing fermented corn gluten feed (FCG) or fermented by-products (FBP) feedstuff.

Items^a^	Diets	
	FCG	FBP
Ingredients, g/kg DM		
Alfalfa hay	300	300
Fermented corn gluten	210	0.00
Fermented bakery by-products	0.00	210
Barley	350	350
Rapeseed meal	97.7	97.7
Slow-release protein	3.50	3.50
NaHCO_3_	3.50	3.50
Xylanase	0.30	0.30
Premix^b^	35.0	35.0
Chemical composition, g/kg DM		
DM	935	940
OM	855	859
CP	158	140
NDF	318	277
EE	44.2	69.5
Starch	133	187
GE, MJ/kg DM	16.3	17.4
NE_m_, MJ/kg DM	6.76	6.81
NE_f_, MJ/kg DM	5.66	5.61

a DM, dry matter; OM, organic matter; CP, crude protein; NDF, neutral detergent fiber; EE, ether extract; GE, gross energy; NE_m_, net energy for maintenance; NE_f_, net energy for fattening. NE_m_ and NE_f_ were calculated.

b The premix (vitamins and microelements) was formulated to provide 250 mg FeSO_4_·7H_2_O, 80 mg CuSO_4_·5H_2_O, 200 mg MnSO_4_·H_2_O, 150 mg ZnSO_4_·H_2_O, 0.5 mg Na_2_SeO_3_, 1 mg KI, 2 mg CoCl_2_·6H_2_O, VA 5000 IU, VD3 3,000 IU, and VE 50 IU in per kg of diet.

### 2.3 Nutrient digestibility

The total fecal output was collected from each bull from d 61 to d 65. Feces were collected immediately after defecation to avoid contamination by urine, hair, feed, or bedding. The feces were thoroughly mixed multiple times a day into a large plastic garbage container with a black garbage bag covering the top to reduce nitrogen losses before subsampling. One subsample (∽1%) was collected and fixed with 10% (w/w) H_2_SO_4_ for CP concentration analyses, and another subsample (∽1%) was collected for analysis of other chemical constituents. The feed provided and any refusals were also weighed and sampled during these 5 consecutive days for the calculation of corresponding feed intake and nutrient digestibility. All samples were dried in a forced oven at 65°C and ground to pass through a 4 mm sieve for subsequent chemical composition analysis.

### 2.4 Rumen contents sampling

Rumen contents were collected from the middle part of the rumen using an oral stomach tube before and 2.5 h after the morning feeding from d 66 to d 67 according to Wang et al. (2016b). To minimize saliva contamination, the initial 50 mL of rumen contents were discarded. The ruminal pH was promptly measured with a pH meter (Starter 300, Ohaus Instruments Co., Ltd., Shanghai, China). Two 35-mL subsamples of rumen contents were placed into a 60-mL syringe, which was connected to a 20-mL syringe via a tube with a length of 2–3 cm. The 20-mL syringe was previously filled with 10 mL nitrogen gas. The nitrogen gas was then injected into the 60-mL syringe through the tube, and the dissolved gasses were extracted into nitrogen in a table concentrator at 200 r/min for 5 min. The extracted gasses were injected into the 20-mL syringe and put into 3.7-mL evacuated tubes for the determination of dissolved hydrogen and dissolved methane.

Another two 10-mL subsamples of rumen contents were promptly frozen and stored at −80°C for subsequent DNA extraction and microbial analysis. Additionally, three 2-mL subsamples of rumen contents were centrifuged at 15,000 g for 10 min at 4°C. Aliquots of the supernatants (1.5 mL) were transferred into plastic tubes each containing 0.15 mL of 25% (w/v) metaphosphoric acid, then stored at −20°C for VFA analysis.

### 2.5 Chemical analysis

The DM (Method 945.15), organic matter (OM) (Method 942.05), CP (Method 954.01), and EE (Method 920.39) were determined according to AOAC (2005). NDF was measured with the inclusion of sodium sulfite and α-amylase according to Van Soest et al. (1991). Gross energy (GE) was determined using an isothermal automatic calorimeter (5E-AC8018; Changsha Kaiyuan Instruments Co., Ltd., Changsha, Hunan). Starch content was determined following the method as described by Karthner and Theurer (1981). The concentration of dissolved hydrogen and dissolved methane were determined and calculated according to Wang et al. (2016a). In detail, concentration of dissolved hydrogen and methane were determined by gas chromatography (Agilent 7890A, Agilent Inc., Palo Alto, CA) using a thermal conductivity and a flame ionization detector, respectively. Hydrogen and methane were separated using a Hayesep Q packed column (2.44 m × 1/8 in. × 2.0 mm ID). Individual VFA was analyzed by gas chromatography (Agilent 7890A, Agilent Inc., Palo Alto, CA) as described by Wang et al. (2014). In detail, after re-centrifuging at 15,000 g, the VFA in the supernatants were measured using gas chromatography. The acids were separated with a DB-FFAP column, and detected with an R flame ionization detector. The carrier gas was nitrogen at a rate of 0.8 mL/min. The analysis was initially isothermal for 2 min at 60°C and then increased to 220°C at a rate of 20°C/min, with a detector temperature of 280°C. VFA were identified and quantified from chromatograph peak areas using calibration with external standards. *R_NH2_* was calculated using the stoichiometric models as described by Wang et al. (2016b).

### 2.6 DNA extraction and microbial analysis

The DNA was extracted using the RBB + C method as modified by Yu and Morrison (2004). The quantity and quality of the extracted DNA were evaluated using an ND-2000 spectrophotometer (NanoDrop Technologies, Wilmington, DE). Illumina sequencing was performed on the NovaSeq platform at Huayu Gene Technology Co. Ltd. The 16S rRNA V3-V4 hyper variable regions were amplified with a 6 bp barcode unique to each sample by using the universal primers (341F: 5′-CCTAYGGGRBGCASCAG-3′, 806R: 5′-GGACTACNNGGGTATCTAAT-3′), each tagged with a unique 6 bp barcode for each sample. After PCR amplification, all amplicon libraries were sequenced and the barcodes and sequencing primers were removed before data processing. Data were analyzed according to the methodology described by Li et al. (2022) to obtain the alpha diversity indexes and taxonomic relative abundance. Beta diversity was analyzed based on Bray–Curtis similarity distances (Bray and Curtis, 1957).

### 2.7 Statistical analysis

The SPSS 22.0 software was employed for data analysis. Data were analyzed using a general linear model with the following model:

$$Y_{ijk}=\mu +D_{i}+B_{j}+e_{ijk}$$

where *μ* is the overall mean; *D_i_* is the fixed effect of dietary treatment (*i* = 2, FCG or FBP diet), *B_j_* is the random effect of Holstein bulls (*j* = 10), and *e_ijk_* is the random error term. When sampling time was included, a linear mixed model was employed with the following model:

$$Y_{\mathrm{ijkm}}=\mu +D_{i}+T_{k}+D_{i}\times T_{k}+A_{j}+e_{\mathrm{ijkm}}$$

where *μ* is the overall mean; *D_i_* is the fixed effect of dietary treatment (*i* = 2, FCG or FBP diet); *T_k_* is the repeated measurement of sampling time (*k* = 2, 0 and 2.5 h relative to the morning feeding); *D_i_* × *T_k_* is the interaction between dietary treatment and sampling time; *A_j_* is the random animal effect (*j* = 10); and *e_ijkm_* is the random error term.

Microbial community composition with relative abundance of bacteria was derived from amplicon data according to Xu et al. (2023), which were analyzed using the Wilcoxon rank-sum test in JMP Pro software (version 16.1.0, SAS Institute Inc., Cary, NC, United States). Statistical significance of effects was declared at *p* < 0.05, and a tendency toward significance was noted for 0.05 ≤ *p* < 0.10.

## 3 Results

The FBP feedstuff contained high levels of EE and starch, and low levels of CP and NDF (Table 1), resulting in significant chemical differences between the FBP and FCG diets (Table 2). Despite similar dry matter intake (DMI) between the bulls fed FBP and FCG diets, those on the FBP diet had higher (*p* < 0.05) intake of EE and starch (Table 3). Compared to the FCG diet, the FBP diet exhibited greater (*p* < 0.05) apparent total-tract digestibility of EE and starch, but had lower (*p* < 0.05) digestibility of CP and NDF (Table 3). The results led to similar DM and OM digestibility between the two dietary treatments (Table 3).

**Table 3:** Feed intake and apparent total-tract digestibility in growing Holstein bulls fed diets containing fermented corn gluten feed (FCG) or fermented by-products (FBP) feedstuff.

Items^a^	Diets		SEM	*p*-value
	FCG	FBP		
Intake, kg/d DM				
DM	7.71	8.30	0.432	0.18
OM	6.63	7.25	0.381	0.12
CP	1.17	1.15	0.060	0.73
NDF	2.45	2.32	0.112	0.27
EE	0.32	0.69	0.046	<0.01
Starch	1.15	1.53	0.093	<0.01
Apparent total-tract digestibility				
DM	0.66	0.65	0.016	0.45
OM	0.69	0.68	0.015	0.25
CP	0.71	0.58	0.021	<0.01
NDF	0.48	0.41	0.026	0.01
EE	0.32	0.58	0.037	0.01
Starch	0.85	0.87	0.008	0.04

a DM, dry matter; OM, organic matter; CP, crude protein; NDF, neutral detergent fiber; EE, ether extract; GE, gross energy.

**Figure 1:**
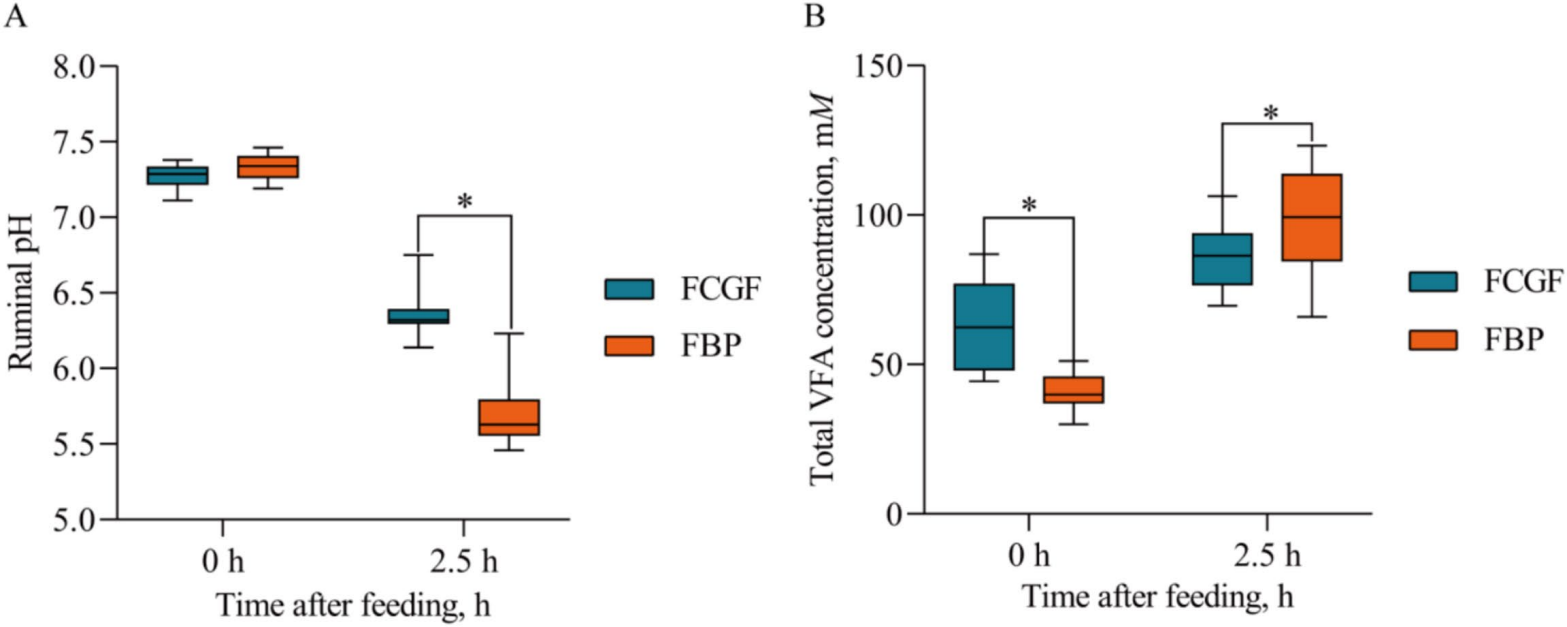
Ruminal pH and total VFA concentration at 0 and 2.5 h after the morning feeding in the rumen of growing Holstein bulls fed with diets containing fermented corn gluten feed (FCG) or fermented by-products (FBP) feedstuff. *Indicates significant difference as *P* < 0.05.

Bulls on the FBP diet had lower (*p <* 0.05) ruminal pH values and dissolved methane concentrations compared to those on the FCG diet (Table 4). Distinct ruminal fermentation profiles were observed between the two diets, with the FBP diet resulting in higher (*p* < 0.05) molar proportions of propionate, and lower (*p* < 0.05) molar proportions of acetate, butyrate, the acetate to propionate ratio, and *R_NH2_* compared to the FCG diet (Table 4). There was a significant interaction effect of diet and sampling time on ruminal pH and total VFA concentration, with greater (*p* < 0.05) total VFA concentration and lower (*p* < 0.05) pH in the FBP diet compared to the FCG diet after the 2.5 h of morning feeding (Figure 1).

**Table 4:** Ruminal dissolved gasses and fermentation end products in growing Holstein bulls fed with diets containing fermented corn gluten feed (FCG) or fermented by-products (FBP) feedstuff.

Items^a^	Diet		Time^b^		SEM	*p*-value		
	FCG	FBP	0 h	+2.5 h		Diet	Time	Diet × time
Dissolved gasses								
Methane, m*M*	0.39	0.28	0.26	0.41	0.046	0.02	<0.01	0.37
Hydrogen, μ*M*	0.77	0.73	0.11	1.39	0.162	0.81	<0.01	0.68
Ruminal pH	6.82	6.52	7.30	6.04	0.053	<0.01	<0.01	<0.01
Total VFA, m*M*	74.6	69.0	51.7	91.9	4.356	0.26	<0.01	<0.01
Molar percentage of individual VFA, mol/100 mol								
Acetate	62.1	55.7	61.2	56.6	0.989	<0.01	<0.01	0.08
Propionate	18.7	28.6	25.6	21.6	1.006	<0.01	<0.01	0.13
Butyrate	15.0	10.1	13.5	11.6	0.561	<0.01	<0.01	0.05
Valerate	1.88	3.09	2.13	2.84	0.163	<0.01	<0.01	0.45
Isobutyrate	0.98	1.05	1.41	0.62	0.064	0.27	<0.01	<0.01
Isovalerate	1.37	1.40	1.97	0.81	0.116	0.84	<0.01	<0.01
Acetate to propionate ratio	3.40	2.03	3.03	2.39	0.142	<0.01	<0.01	0.82
*R_NH2_*	1.34	1.02	1.24	1.12	0.036	<0.01	<0.01	0.21

a VFA, volatile fatty acids; R_NH2_, estimated net H_2_ production relative to the amount of total VFA produced.

b Time, sampling time after the morning feeding.

**Figure 2:**
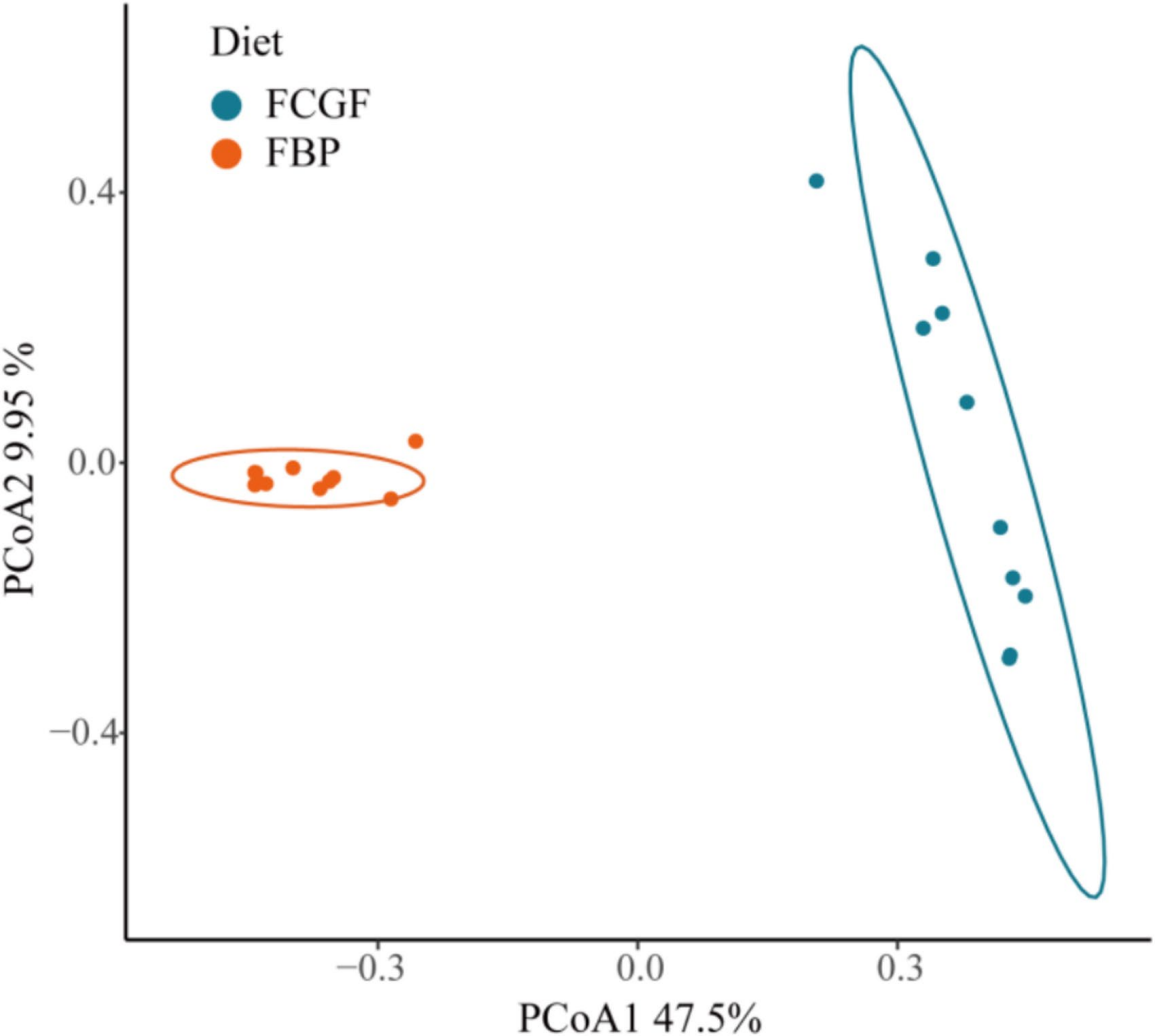
PCoA analyses of bacterial communities in the rumen of growing Holstein bulls fed with diets containing fermented corn gluten feed (FCG) or fermented by-products (FBP) feedstuff (*p* = 0.001, *R*^2^ = 0.46).

**Figure 3:**
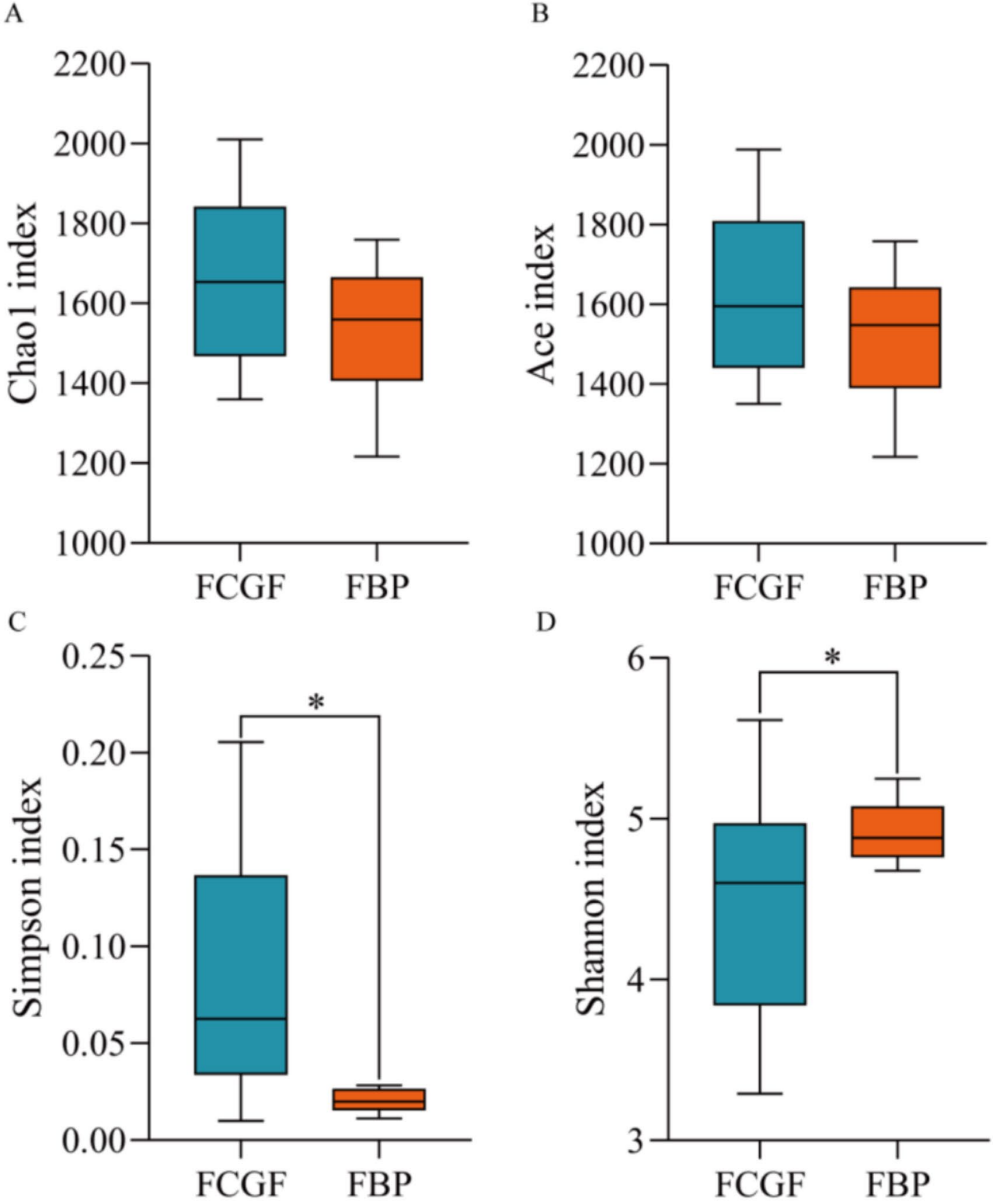
Alpha diversity indices of bacteria community in the rumen of growing Holstein bulls fed with diets containing fermented corn gluten feed (FCG) or fermented by-products (FBP) feedstuff. *Indicates significant difference as *P* < 0.05.

Bulls on the FBP diet had distinct ruminal bacterial communities compared to those on the FCG diet, as samples were clearly separated by PC1 (explaining 47.5% of the variation) (*p* < 0.05, Figure 2). Feeding the FBP diet enhanced bacterial diversity, as indicated by the greater (*p* < 0.05) Shannon index and lower Simpson index compared to feeding FCG diet (Figure 3). A total of 16 phyla were identified with Bacteroidetes and Firmicutes being the most abundant (Table 5). Bulls on the FBP diet had a greater (*p* < 0.05) ruminal relative abundance of Firmicutes, Proteobacteria and Actinobacteriota, and a lower (*p* < 0.05) abundance of Bacteroidetes compared to those on the FCG diet. In total, 219 genera were identified, with *Prevotella* being the most abundant (Table 5). The FBP diet resulted in a greater (*p* < 0.05) relative abundance of *Succiniclasticum*, *Lachnospiraceae_unclassified*, *Selenomonadaceae_unclassified*, *Lachnospiraceae_NK3A20_group*, *Megasphaera*, and *Prevotellaceae_unclassified*, and a lower (*p* < 0.05) abundance of *Rikenellaceae_RC9_gut_group, F082_unclassified, Christensenellaceae_R-7_group and Ruminococcus* compared to the FCG diet (Table 5).

**Table 5:** The relative abundance (>1%) of bacteria in the rumen of growing Holstein bulls fed diets containing fermented corn gluten feed (FCG) or fermented by-products (FBP) feedstuff.

Items	Diets		SEM	*p*-value
	FCG	FBP		
Phylum Bacteroidota	71.0	39.7	4.003	<0.01
*Prevotella*	46.6	30.6	5.056	<0.01
*Rikenellaceae_RC9_gut_group*	12.4	1.04	1.578	<0.01
*F082_unclassified*	5.46	0.58	0.676	<0.01
*Prevotellaceae_unclassified*	1.93	4.11	1.003	0.04
*Prevotellaceae_UCG-003*	1.52	0.29	0.242	<0.01
*Bacteroidales_RF16_group_unclassified*	1.26	0.09	0.246	<0.01
*Prevotellaceae_UCG-001*	0.73	1.26	0.355	0.15
Phylum Firmicutes	25.9	50.5	4.516	<0.01
*Succiniclasticum*	4.99	8.69	1.458	0.02
*Lachnospiraceae_unclassified*	1.01	5.07	1.213	<0.01
*Clostridia_UCG-014_unclassified*	2.57	3.65	0.912	0.25
*Christensenellaceae_R-7_group*	3.71	0.26	0.846	<0.01
*Lachnospiraceae_NK3A20_group*	0.67	4.00	0.816	<0.01
*Megasphaera*	0.00	3.59	0.841	<0.01
*Ruminococcus*	3.09	0.54	0.532	<0.01
*NK4A214_group*	2.66	0.68	0.701	0.01
*Selenomonadaceae_unclassified*	0.43	2.39	0.603	<0.01
*Shuttleworthia*	0.02	2.12	1.016	0.05
*Lachnospiraceae_unclassified*	0.14	1.91	0.373	<0.01
*Acidaminococcus*	0.00	1.65	0.228	<0.01
*Ruminococcaceae_unclassified*	1.43	0.04	0.316	<0.01
*Acetitomaculum*	0.25	1.24	0.511	0.06
*Lachnospira*	0.08	1.21	0.272	<0.01
*Oribacterium*	0.07	1.20	0.236	<0.01
*Oscillospirales_unclassified*	1.00	1.17	0.368	0.63
Phylum Proteobacteria	0.30	3.50	1.388	0.03
*Succinivibrionaceae_UCG-001*	0.00	2.80	1.013	0.01
Phylum Actinobacteriota	0.58	3.44	0.781	0.02
*Olsenella*	0.28	1.83	0.532	<0.01
*Pseudoscardovia*	0.01	1.19	0.283	<0.01
Phylum Patescibacteria	1.03	0.83	0.286	0.49
*Candidatus_Saccharimonas*	1.26	0.42	0.349	0.02
Others	1.21	2.07	0.323	0.02
*Others*	10.4	16.4	2.366	0.02

## 4 Discussion

Although replacing FCG with FBP feedstuff had little influence on nutrient intake, it increased the intake and digestibility of EE and starch, and decreased CP and NDF digestibility. The reduction of NDF digestibility could be attributed to the greater inclusion of starch or fat, as these components are known to negatively affect ruminal fibrolytic bacteria and protozoa, thus impairing fiber digestibility (NRC, 2001; Patra, 2013; Bhatt et al., 2011). Furthermore, the heating process of bakery by-products can activate the Maillard reactions of CP, hindering ruminal CP degradability (Purlis, 2010), which results in lower CP digestibility in FBP dietary treatment. These distinct changes in the digestibility of starch, EE, NDF, and CP contributed to the similar total feed digestibility of DM observed between the two dietary treatments.

Methane is a major end product for methanogenesis during the rumen fermentation of ingested carbohydrates (Wang et al., 2015). Carbohydrates with higher starch content degrade more rapidly in the rumen, often resulting in a rapid increase in ruminal dissolved methane concentration (Wang et al., 2016b). However, it is not the case in present study. Lower dissolved methane concentrations were observed despite higher starch intake in the FBP diet, along with a decreased pH value at 2.5 h after the morning feeding. A rapid pH drop below 6.0 can inhibit ruminal methanogenesis, as the activities of methanogens decrease at pH below 6.0 (Van Kessel and Russell, 1996; Qiu et al., 2023). Moreover, the increased EE intake with the FBP diet likely contributed to the reduced ruminal methanogenesis. Bakery by-products are rich in EE with greater percentages of unsaturated fatty acids (Humer et al., 2018), which have been reported to have negative effects on methanogens by adhering to the surface or smearing the cell walls and thus impeding the essential nutrient passage (Henderson, 1973; Prins et al., 1972; Van Nevel and Demeyer, 1996). Therefore, the reduced ruminal methanogenesis could be attributed to the combined effects of lower ruminal pH and higher EE intake after replacing FCG with FBP feedstuff.

Hydrogen is formed during the ruminal fermentation of carbohydrates into VFA, and is primarily utilized by archaeal methanogens to form methane (Janssen, 2010; van Lingen et al., 2016). When methanogen activity is inhibited, hydrogen partial pressure accumulates in the rumen (Van Kessel and Russell, 1996; Wang et al., 2013). However, the dissolved hydrogen concentration remained unchanged despite the inhibition of ruminal methanogenesis in the FBP diet. It could be due to the reduced hydrogen accumulation, which is determined by the balance of hydrogen production and utilization (Wang et al., 2016b). The reduced rumen pH and increased EE intake likely inhibited the growth of protozoa (Janssen, 2010), which are important hydrogen producers in the rumen microbial ecosystem (Muller, 1980; Paul et al., 1990), leading to reduced hydrogen accumulation in the FBP diet.

Starch fermentation favors propionate production over acetate production, while fiber fermentation produces more acetate than propionate (Bannink et al., 2006; Song et al., 2018). Propionate synthesis serves as a hydrogen sink whereas acetate and butyrate production are accompanied by net hydrogen generated (Janssen, 2010; Ungerfeld, 2015; Li et al., 2024). The FBP diet showed higher molar percentages of propionate and lower molar percentages of acetate and butyrate. Increased propionate production indicates reduced hydrogen produced per mole of VFA, as is consistent with the reduced *R_NH2_* observed. The decreased efficiency of hydrogen production aligns with the reduced ruminal methanogenesis in the FBP diet and may partially explain the unchanged dissolved hydrogen concentration.

Furthermore, the introduction of FBP feedstuff into the diet likely alters the fermentation dynamics in the rumen, which in turn could influence microbial populations. Specifically, the high starch content in the FBP diet leads to a decrease in ruminal pH, creating an environment more favorable to starch-degrading bacteria. The shift in microbial community composition could increase populations of Succiniclasticum species, which are involved in the fermentation of starch and production of succinate (Bi et al., 2018; Cancino-Padilla et al., 2021). Additionally, the abundance of Megasphaera, which plays a major role in the production of propionate, is likely to increase (Pitta et al., 2014; Humer et al., 2018).

Such a shift in microbial community could result in a reduction in hydrogen availability for methanogenic archaea, thereby altering hydrogen metabolism in rumen. *Prevotella*, known for degrading wide ranges of proteins, starch, hemicellulose and pectin to produce VFA and acting as a hydrogen consumer (Mickdam et al., 2017; Betancur-Murillo et al., 2022), was unexpectedly reduced in the FBP diet, possibly due to high levels of fat in the FBP diet (Cremonesi et al., 2018). Further studies are needed to understand the role of *Prevotella* on ruminal lipid metabolism. Instead, *Succiniclasticum* and *Megasphaera* were enriched in the rumen of bulls on the FBP diet. Oil supplementation and elevated feed energy levels have been reported to promote the growth of *Succiniclasticum*, which converts succinate to propionate, providing an alternative pathway of hydrogen utilization for propionate formation (Bi et al., 2018; Cancino-Padilla et al., 2021). *Megasphaera*, a major soluble sugar fermenter, utilizes lactate (almost up to 80%) to produce propionate and is therefore boosted by diets high in digestible starch or sugar (Pitta et al., 2014; Humer et al., 2018). Thus, the enrichment of *Succiniclasticum* and *Megasphaera* likely promoted hydrogen incorporation for propionate synthesis in the FBP dietary treatment.

Cellulose-degrading bacteria are major producers of acetate with net hydrogen production (Chaucheyras-Durand et al., 2010; Pang et al., 2022). Feeding the FBP diet had lower abundance of fibrolytic bacteria, such as *Christensenellaceae_R-7_group*, *Ruminococcus* and *NK4A214_group*, which was consistent with lower ruminal acetate percentage and *R_NH2_*. Biohydrogenating bacteria, such as *Lachnospiraceae_unclassified* and *Lachnospira* (Boeckaert et al., 2008), were greatly enriched in FBP dietary treatment, which was consistent with its elevated EE intake with enhancement of net hydrogen incorporation.

## 5 Conclusion

Replacing FCG with FBP increases EE and starch digestibility but decreases NDF and CP digestibility, with minimal impact on DM or OM digestibility. The increase in starch and EE intakes after replacing FCG by FBP feedstuff decreases ruminal methanogenesis by shifting rumen fermentation from acetate to propionate production through enriching *Succiniclasticum*, *Megasphaera*, *Lachnospiraceae_unclassified*, and *Lachnospira* which could reduce the reliance on hydrogen for methane synthesis by methanogenic archaea, and inhibiting *Christensenellaceae_R-7_group*, *Ruminococcus* and *NK4A214_group*. Consequently, FBP feedstuff can serve as an effective alternative to FCG with the additional benefit for inhibiting methanogenesis in the rumen.

## Data Availability

The original contributions presented in the study are publicly available. This data can be found here: https://www.ncbi.nlm.nih.gov/bioproject/PRJNA1151939.
